# Aviator Occupational Behavior Surrounding COVID-19 Infection and Vaccination in the United States: A Cross-Sectional Population-Based Survey

**DOI:** 10.7759/cureus.23406

**Published:** 2022-03-22

**Authors:** William R Hoffman, James Aden, Joshua D Luster

**Affiliations:** 1 Department of Neurology, Brooke Army Medical Center, San Antonio, USA; 2 Department of Graduate Medical Education, Brooke Army Medical Center, San Antonio, USA

**Keywords:** international and travel medicine, vaccination policy, aviation, covid-19, aerospace medicine

## Abstract

Introduction

The coronavirus disease 2019 (COVID-19) pandemic has precipitated change across the aviation industry, including aeromedical standards. U.S. pilot occupational behavior regarding COVID-19 infections and vaccinations have not been well-studied.

Methods

We conducted an anonymous survey of 661 U.S. pilots from September 1, 2021, through December 15, 2021.

Results

We found 23.8% of pilots reported a history of COVID-19 infection but only 20.5% of infected pilots reported this history to an aeromedical examiner (AME)/flight surgeon. Of uninfected pilots, 50.5% reported being either extremely unlikely or somewhat unlikely to disclose a new infection to an AME/flight surgeon. Seventy-nine point six percent (79.6%) of pilots received at least one dose of any COVID-19 vaccine and 89.6% of those who received a vaccine complied with the 48-hour no-flying policy. Of the unvaccinated pilots, 74.5% reported being either extremely unlikely or somewhat unlikely to receive a vaccine.

## Introduction

The coronavirus disease 2019 (COVID-19) pandemic has dramatically changed healthcare behaviors across the world, including aeromedical standards in the United States (U.S.) [1]. In the U.S., military pilots and certain civilian pilots are required to undergo regular aeromedical screening to ensure mandated standards (tiered based on flying duties) are met [2-3]. If a pilot develops a new symptom or condition and is unable to meet these standards, the pilot runs the risk of temporarily or permanently losing their aeromedical certificate. COVID-19 infection has a spectrum of illnesses, ranging from an absence of symptoms to intensive care unit (ICU) level medical needs. Further, a subset of patients reports chronic symptoms persisting beyond the period of acute infection [4]. This spectrum of disease raises questions about aeromedical disposition, including a return to flying duty. The subsequent approval of multiple COVID-19 vaccines raised similar questions. At the time of writing, the current U.S. Federal Aviation Administration (FAA) guidelines direct civilian pilots to disclose a COVID-19 infection during aeromedical screening but permits an aeromedical examiner (AME) to issue an aeromedical certificate unless the pilot required an ICU admission or the pilot reported residual or prolonged symptoms beyond the acute infection [5]. Further, current FAA guidance precludes pilots from functioning as a required aircrew member within 48 hours of vaccination [6]. Given the risk of permanent or temporary aeromedical certificate loss in the face of COVID-19 infection and pilot healthcare avoidance demonstrated in other studies [7-9],^ ^we hypothesized that a proportion of pilots may not comply with current guidance. To our knowledge, pilot behavior surrounding COVID-19 infection and vaccination has not been well-studied and has limited reference in the medical literature. The objectives of the current study were to study pilot behavior (1) surrounding COVID-19 infection, (2) surrounding COVID-19 vaccination, and to (3) study rates of compliance regarding FAA COVID-19 infection and vaccination guidelines. The intention of this study was to better understand pilot healthcare behavior related to COVID-19 vaccination and reporting.

## Materials and methods

Study design

The researchers conducted this cross-sectional study in accordance with the Declaration of Helsinki per the Brooke Army Medical Center Institutional Review Board approved study protocol (protocol no. C.2019.158e). The study used a non-probabilistic internet survey of the general population of U.S. pilots. The survey was hosted using Qualtrics software (Qualtrics XM, Seattle, WA) and was accessible by respondents via an URL that was active during the period of September 1, 2021, through December 15, 2021. The inclusion criteria were any individual holding an appropriate certification granting authority to operate an aircraft in the U.S.

The study was publicized on social media platforms, including Instagram.com and Facebook.com, by searching for aviation-related interest groups using the following keywords: pilots, aviation, student pilots, flying, and airline. Additionally, “Pilots’ Pandemic,” a large aviation interest podcast, advertised the study on several episodes as well as on the associated social media sites. The researchers supplemented the primary social media advertisement campaign by advertising the study through the following communication channels: e-mail lists and social media groups affiliated with pilots employed by one U.S. airline. The sample was collected during the surge of the Omnicron variant of COVID-19 in the United States and the authors elected to conclude the study on December 15, 2021, to complete data analysis. No sample size calculations were conducted prior to the study.

Questionnaire

The survey included 13 questions in English only and collected no personally identifiable information. Based on the limited prior research related to this topic, the authors were unable to identify a previously used or independently validated set of survey questions. The studied questions were generated by two resident physicians (one a certified FAA AME with both physicians caring for pilots and non-pilots in the United States). Two items addressed respondent demographic factors: age (<25, 25-40, 41-60, or >60) and gender (male, female, or other). Responders were asked their predominant type of flying during the past five years (military, jet; military, transport; military, other; civilian, mainline airline transport; civilian, regional airline transport; civilian, non-airline commercial; and civilian, general aviation). All participants were then asked whether they had ever been diagnosed (clinically or laboratory data) with COVID-19 infection. If the participant answered yes, they were then asked (1) if they had disclosed this diagnosis to an AME/flight surgeon, (2) if they experienced symptoms they felt were related to COVID-19 infections that lasted longer than 14 days, and (3) whether they had operated an aircraft prior to evaluation by an AME/flight surgeon. If the participant answered no to ever being infected with COVID-19, they were asked how likely they would be to disclose a new COVID-19 infection to an AME/flight surgeon using the Likert scale (where 1 = extremely unlikely and 5 = extremely likely). All participants were then asked whether they had received at least one COVID-19 vaccine dose. Participants who answered yes were then asked (1) if they had functioned as a required aircrew member within 48 hours and (2) whether they had experienced a symptom they felt was related to the COVID-19 vaccine that impacted their ability to perform their flying duties. If the participant responded that they had not received at least one dose of a COVID-19 vaccine, they were asked how likely they would be to undergo vaccination using a Likert Scale (where 1 = extremely unlikely and 5 = extremely likely).

Data analysis

Surveys were excluded if (1) the responder did not agree to the informed consent question and/or (2) two or fewer demographic questions were unanswered. Pilot types were pooled into three categories that included the following subgroups: (1) civilian paid (civilian, mainline airline transport; civilian, regional airline transport; civilian, non-airline commercial), (2) civilian nonpaid (civilian, general aviation), and (3) military (military jet; military transport; military other). The 41-60 and >60 years old age categories were pooled together due to small enrollment numbers. Categorical data were summarized using percentages and analyzed using the chi-squared test. Means and standard deviations were used as summary statistics for the Likert-type scale and ordinal variables and were analyzed using Wilcoxon’s and Kruskal-Wallis tests. Significance for results was established when p-values were less than 0.05. Odds ratios and their corresponding 95% confidence interval and p-values were then reported for all factors in the model. All statistical analysis was performed using JMP v 13.2 SAS Corp (Cary, NC).

## Results

Responses from 715 participants were reviewed, and 661 ultimately met the inclusion criteria (14 responders did not agree to the informed consent question and 40 responders answered two or fewer demographic questions).

**Figure 1:**
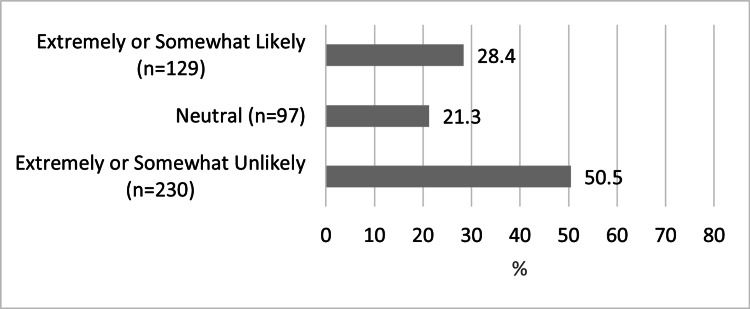
Pilot Likelihood to Disclose New Diagnosis of COVID-19 Infection

Table 1 shows the responses for questions regarding COVID-19 infection and related health behavior by demographic factors. Table 2 shows the responses for questions regarding COVID-19 vaccination and related health behavior by demographic factors. Figure 1a shows the reported likelihood to disclose a new COVID-19 infection to an AME/flight surgeon in pilots with no prior history of COVID-19 infection, and Figure 1b shows the likelihood of undergoing COVID-19 vaccination in pilots with no history of prior COVID-19 vaccination.

**Table 1:** Pilot Operational Behavior Surrounding COVID-19 Infection

	Yes (%)	No (%)	p
Diagnosed with COVID-19 (Lab Test or Clinical)	152 (23.8)	486 (76.2)	
Males	142 (25.0)	427 (75.0)	
Female	10 (15.4)	55 (84.6)	0.074
Civilian, Paid	60 (24.8)	182 (75.2)	
Civilian, Non-Paid	80 (22.3)	279 (77.7)	
Military	12 (32.4)	25 (67.6)	0.367
<25 years old	70 (22.7)	238 (77.3)	
26-40 years old	73 (25.6)	212 (74.4)	
≥41 years old	9 (20.0)	36 (80.0)	0.583
Reported COVID-19 Diagnosis to AME/Flight Surgeon	30 (20.5)	116 (79.5)	
Males	28 (20.6)	108 (79.4)	
Female	2 (20.0)	8 (80.0)	0.964
Civilian, Paid	16 (27.1)	43 (72.9)	
Civilian, Non-Paid	8 (10.7)	67 (89.3)	
Military	6 (50.0)	6 (50.0)	0.003
<25 years old	8 (11.9)	59 (88.1)	
26-40 years old	20 (28.6)	50 (71.3)	
≥41 years old	2 (22.2)	7 (77.8)	0.05
COVID-19 Symptoms Lasting Longer than 14 days	31 (21.2)	115 (78.8)	
Males	28 (20.6)	108 (79.4)	
Female	3 (30.0)	7 (70.0)	0.5
Civilian, Paid	12 (20.3)	47 (79.7)	
Civilian, Non-Paid	12 (16.0)	63 (84.0)	
Military	7 (58.3)	5 (41.7)	0.01
<25 years old	9 (13.9)	56 (86.1)	
26-40 years old	19 (26.4)	53 (73.6)	
≥41 years old	3 (33.3)	6 (66.7)	0.127
Flew Prior to Evaluation by AME/Flight Surgeon	82 (58.2)	59 (41.8)	
Males	79 (59.9)	53 (40.2)	
Female	3 (33.3)	6 (66.7)	0.121
Civilian, Paid	34 (59.7)	23 (40.4)	
Civilian, Non-Paid	42 (58.3)	30 (41.7)	
Military	6 (50.0)	6 (50.0)	0.828
<25 years old	32 (51.6)	30 (48.4)	
26-40 years old	44 (62.0)	27 (38.0)	
≥41 years old	6 (75.0)	2 (25.0)	0.287

**Table 2:** Pilot Operational Behavior Surrounding COVID-19 Vaccination

	Yes (%)	No (%)	p
Received at Least One Dose of COVID-19 Vaccine	471 (79.6)	121 (20.4)	
Males	423 (79.8)	107 (20.2)	
Females	45 (77.6)	13 (22.4)	0.693
Civilian, Paid	180 (80.7)	43 (19.3)	
Civilian, Non-Paid	260 (78.3)	72 (21.7)	
Military	31 (83.8)	6 (16.2)	0.629
<25 years old	217 (77.2)	64 (22.8)	
26-40 years old	223 (82.6)	47 (17.4)	
≥41 years old	31 (75.6)	10 (24.4)	0.237
Flew Within 48 Hours of COVID-19 Vaccination	48 (10.4)	414 (89.6)	
Males	42 (10.2)	371 (89.8)	
Females	6 (13.0)	40 (87.0)	0.558
Civilian, Paid	26 (14.7)	151 (85.3)	
Civilian, Non-Paid	16 (6.3)	238 (93.7)	
Military	6 (19.4)	25 (80.7)	0.005
<25 years old	18 (8.5)	193 (91.5)	
26-40 years old	26 (11.8)	194 (88.2)	
≥41 years old	4 (12.9)	27 (87.1)	0.475
Experienced a Symptom After Vaccine that Reportedly Impacted Ability to Perform Flying Duties	80 (17.9)	368 (82.1)	
Males	66 (16.5)	334 (83.5)	
Females	14 (31.1)	31 (68.9)	0.023
Civilian, Paid	30 (17.7)	140 (82.4)	
Civilian, Non-Paid	43 (17.5)	203 (82.5)	
Military	7 (22.6)	24 (77.4)	0.79
<25 years old	39 (19.1)	165 (80.9)	
26-40 years old	37 (17.4)	176 (82.6)	
≥41 years old	4 (12.9)	27 (87.1)	0.667

**Figure 2:**
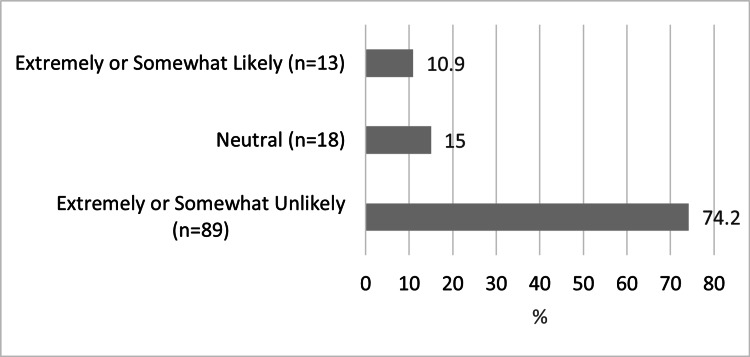
Unvaccinated Pilot Self-Reported Likelihood to Receive COVID-19 Vaccination

## Discussion

The objective of the current study was to examine U.S. pilot behavior surrounding both COVID-19 infection and vaccination, in addition to compliance rates with related guidelines. To our knowledge, this study appears to be among the first to investigate pilot behavior surrounding COVID-19 infection and vaccination in the United States. Our sample is a limited representation of civil pilots in the United States by age and subtype with an under-representation of civilian pilots >41 years old and military pilots [10]. The sample’s gender distribution is a limited representation of the civil aviator population [10]^ ^though our study was underpowered to confidently say whether the sample is representative of military pilots by gender [11].

In our cohort, 23.8% of pilots had been diagnosed with COVID-19 infection (n=152), which is likely lower than the general population based on available data [12]. Despite this difference, our cohort’s infection rate based on pilot age had a similar distribution to the general population, where younger pilots trended towards accounting for more infections compared to older pilots [13]. Of the pilots who reported a history of infection, only 20.5% reported disclosing this history to an AME/flight surgeon (n=30). This finding could have several explanations, including a pilot not yet requiring aeromedical screening, delayed aeromedical screening due to the pandemic [14], or pilot healthcare anxiety [7-9]. There was a significant difference (p = 0.003) between pilot subtypes, where paid civilian and military pilots disclosed a previous infection at higher rates than non-paid pilots. This may be related to increased mandatory aeromedical screening frequency in these populations. There were 21.2% of pilots in our cohort who reported symptoms that they thought were related to COVID-19 infection beyond 14 days (n=31), which is a circumstance that requires further aeromedical evaluation prior to aeromedical certificate issuance [5]. Military pilots were more likely to report this than civilian pilots though the clinical significance of this is uncertain. Interestingly, there were 58.2% of pilots in our cohort who reported flying prior to evaluation by an AME/flight surgeon after infection (n=82). This finding could be related to an infection course being perceived as aeromedically insignificant by the pilot, limited AME/flight surgeon access during the pandemic [14], or pilot healthcare-seeking anxiety [7-9] among other explanations. The possibility of pilot healthcare-seeking anxiety may be supported by the 50.5% of pilots in our cohort with no prior COVID-19 infection (n=230) who reported being either extremely unlikely or somewhat unlikely to disclose a new infection of COVID-19.

The vast majority of pilots in our cohort had received at least one dose of a COVID-19 vaccine (79.6%, n=471), which nearly mirrors that of the United States as of the time of writing [15]. Interestingly, 89.6% of pilots in our cohort reported compliance with the FAA’s rule of no flying within 48 hours of vaccination, a rule not universal around the world [16]. A minority of pilots in our cohort reported experiencing a symptom after vaccination that impacted their ability to fly (17.9%, n=80), where female pilots were more likely to report this than males. We did not study whether these pilots went on to seek aeromedical evaluation due to these symptoms or the aeromedical implications of this finding. Of the pilots who had not received at least one vaccine dose, 74.2% reported that they were either extremely unlikely or somewhat unlikely to receive a vaccine (n=89), a finding reported of other populations [17]. To our knowledge, this data is the first reported on aviator COVID-19 vaccine hesitancy in the United States.

This study has important limitations. Retrospective studies are at risk for recall bias, and non-probability sampling may lead to response bias. Further, the relative underrepresentation of civilian pilots >41 years old and military pilots may have impacted the results. It is possible that the use of social media as a recruiting method may have contributed to higher participation in younger pilots relative to older pilots. No answers were able to be verified, and it is not known how a diagnosis of COVID-19 infection was made for any participant. A response rate cannot be calculated because it is unknown how many pilots received an invitation to participate. Several of our study questions were about dynamic population health information, and the results will likely evolve as the pandemic continues. Importantly, we are not able to say whether these data portend an increased risk to aviation safety. We feel these data are largely hypotheses-generating and primarily argue for further research. Despite these limitations, the present study is a contribution to the limited available data on pilot healthcare behavior related to COVID-19 infection and vaccination.

## Conclusions

A vast majority of pilots reported receiving at least one dose of the COVID-19 vaccine, but the unvaccinated cohort reported a low likelihood to undergo vaccination. A subset of U.S. pilots may not report a history of COVID-19 infection to an AME/flight surgeon, and a subset of unvaccinated U.S. pilots may experience vaccine hesitancy. In our cohort, most pilots reported receiving at least one dose of a COVID-19 vaccine and following related U.S. no-flying guidance. Future research should focus on the aeromedical implications of incomplete health information reporting and vaccine hesitancy.
